# An international internet survey of the experiences of 1,714 mothers with a late stillbirth: the STARS cohort study

**DOI:** 10.1186/s12884-015-0602-4

**Published:** 2015-08-15

**Authors:** Jane Warland, Louise M. O’Brien, Alexander E. P. Heazell, Edwin A. Mitchell, the STARS consortium

**Affiliations:** Mothers, Babies and Families: Health Research Group, School of Nursing and Midwifery University of South Australia, Adelaide, SA Australia; Sleep Disorders Center, Department of Neurology, Department of Obstetrics and Gynecology, and Department of Oral and Maxillofacial Surgery, University of Michigan, Ann Arbor, MI USA; Maternal and Fetal Health Research Centre, University of Manchester, Manchester, UK; Department of Paediatrics: Child and Youth Health, University of Auckland, Auckland, New Zealand

## Abstract

**Background:**

Stillbirth occurring after 28 weeks gestation affects between 1.5–4.5 per 1,000 births in high-income countries. The majority of stillbirths in this setting occur in women without risk factors. In addition, many established risk factors such as nulliparity and maternal age are not amenable to modification during pregnancy. Identification of other risk factors which could be amenable to change in pregnancy should be a priority in stillbirth prevention research. Therefore, this study aimed to utilise an online survey asking women who had a stillbirth about their pregnancy in order to identify any common symptoms and experiences.

**Methods:**

A web-based survey.

**Results:**

A total of 1,714 women who had experienced a stillbirth >3 weeks prior to enrolment completed the survey. Common experiences identified were: perception of changes in fetal movement (63 % of respondents), reports of a “gut instinct” that something was wrong (68 %), and perceived time of death occurring overnight (56 %). A quarter of participants believed that their baby’s death was due to a cord issue and another 18 % indicated that they did not know the reason why their baby died. In many cases (55 %) the mother believed the cause of death was different to that told by clinicians.

**Conclusions:**

This study confirms the association between altered fetal movements and stillbirth and highlights novel associations that merit closer scrutiny including a maternal gut instinct that something was wrong. The potential importance of maternal sleep is highlighted by the finding of more than half the mothers believing their baby died during the night. This study supports the importance of listening to mothers’ concerns and symptoms during pregnancy and highlights the need for thorough investigation of stillbirth and appropriate explanation being given to parents.

**Electronic supplementary material:**

The online version of this article (doi:10.1186/s12884-015-0602-4) contains supplementary material, which is available to authorized users.

## Background

In high-income countries the stillbirth rate ≥28 weeks ranges between 1.5 and 4.5 per 1,000 births and has remained fairly consistent over the last two decades [1, 2]. It is notable that there is considerable disparity in the rates of stillbirth in different high-income countries ranging from 1.5 per 1,000 total births in the Czech Republic to 4.3 per 1,000 total births in France [1]. The reasons for these differences are not understood but the disparity suggests that those with higher rates may be able to be improved [2]. In 2011, the Lancet Stillbirth Series called for high-income countries to eliminate all preventable stillbirths and close equity gaps by 2020 [3], to achieve this further research is needed to identify factors that may prevent stillbirth [2].

### Risk factors for stillbirth

Modifiable, and or potentially modifiable, risk factors for stillbirth include maternal age (>35 years), obesity (BMI >30 kg/m^2^), and cigarette smoking [4]. Pre-existing diabetes, chronic hypertension, substance misuse, black race, and nulliparity are commonly cited associations with stillbirth [5]. However, these risk factors known at the onset of pregnancy only account for a small proportion (19 %) of stillbirths [6]. Therefore, the ability to predict and prevent stillbirth remains poor, as most stillbirths occur in women who are deemed to be at “low-risk” of pregnancy complications. Importantly, with the exception of control of maternal disease and substance misuse, few of these established risk factors are amenable to modification during pregnancy. Therefore, if improvements are to be made in stillbirth prevention, specific modifiable factors must be identified and targeted.

We aimed to investigate potentially modifiable risk factors in a large, international population. In contrast to the majority of previous studies that have investigated social or demographic associations with stillbirth, we planned to meet this aim by developing an approach that asked mothers of stillborn babies directly about their behaviours, experiences, and symptoms during pregnancy. To achieve this goal an international group of researchers and clinicians, the STARS (Study of Trends and Associated Risks for Stillbirth) Consortium, partnered with the Star Legacy Foundation and other stillbirth and parental support groups to conduct a web-based survey of women who had experienced a stillbirth using a nested case–control design with an uncontrolled cohort. Here we present the findings from 1,714 stillbirth cases in the cohort arm of the study.

## Subjects and methods

### Methods

Women aged at least 18 years of age who had experienced a singleton stillbirth (≥28 weeks) more than 3 weeks previously, were eligible to participate. There was no upper limit as to how long ago the stillbirth occurred.

### Survey design

This international, anonymous, web-based study was developed during the inaugural Stillbirth Summit in October 2011 [7]. The STARS consortium was formed between several clinicians, academics, researchers, and bereaved parents from Australia, New Zealand, the United Kingdom and the United States of America. This unique partnership allowed bereaved parents to have direct discussions with the consortium members regarding common experiences prior to their loss in order to inform the development of the survey.

The survey included questions related to established risk factors (e.g. cigarette smoking, perceived changes in fetal movements) as well as questions relating to emerging risk factors (e.g. gut instinct that something was wrong during the pregnancy and an increase in fetal activity in the days immediately prior to the fetal demise). Several questions included in the survey were raised by bereaved parents at the 2011 Stillbirth Summit and have not been previously addressed in large-scale studies.

An online survey was constructed in line with the principles for web survey design proposed by Dillman [8]. A web-based survey format was chosen in order to increase the ease with which the survey could be widely distributed, to allow participants easy access to the survey and to reduce the costs of conducting an international study. The survey was reviewed by consortium members who had experience in the conduct of surveys and revisions were made based upon their feedback. To ensure that the questions would solicit targeted information and that the time taken to complete the survey was not too onerous, the survey was piloted with a group of bereaved mothers (*n* = 6) accessed through the Star Legacy Foundation.

Following this pilot phase, minor alterations were made to the survey and it was launched in September 2012. The survey included open-ended responses, categorical responses, such as yes/no/don’t know, Likert scales, and selection of responses from a list either through drop-down menus that allowed single responses or check boxes that allowed multiple responses. The format of the final survey included branching logic such that participants were directed through different paths based on their response. There were no compulsory questions such that participants were allowed to skip questions if they wished. The questionnaire is available as a supplementary file to the manuscript. In reporting this study, guidelines from strengthening the Reporting of Observational Studies in Epidemiology (STROBE) group were followed.

### Ethical approval

This study was approved by the Institutional Review Board (IRB) of the University of Michigan (HUM#00063655). Prior to gaining access to the survey participants were informed about the purpose of the study (to look for trends and risk-factors for stillbirth) as well as contact information for a stillbirth support group (First Candle) if they became distressed whilst completing the survey. Informed consent was gained by the participant clicking an “I agree” button prior to gaining access to the survey.

### Participants

Participants were recruited to this study by international web-based advertising, social media, and word of mouth between September 2012 and August 2014. All participants completed the survey, which asked about their recollection of their own activity, fetal activity and maternal perception of cause of death (COD). No identifying details were collected. Women could elect to provide their email addresses if they wished to receive a copy of the published results. However, this was not a requirement of participation. If they gave their email address then it was stored separately from survey responses.

### Inclusion criteria

Women, at least 18 years of age, who were fluent in reading and writing English and who had a history of a singleton stillbirth at or greater than 28 weeks gestation were invited to complete the online survey.

### Exclusion criteria

Participants with multiple gestation pregnancies, neonatal death, or fetal loss prior to 28 weeks gestation were excluded.

### Analysis

The analysis for this study involved simple descriptive statistics. The numerical results are expressed in terms of frequencies and proportions. Comparisons between sub-groups were made using Chi-Square tests with statistical significance set at *p* ≤ 0.05. Qualitative text response data, such as cause of death (COD) and description of changes in fetal movements, were coded by two investigators (JW and LMO) into dichotomized variables to determine frequencies of responses. For COD, data regarding what women were told vs. what women believed was the COD were first coded by JW who has substantial experience in this type of coding. This coding was then checked by LMO and where discrepancies were raised these were discussed and a consensus arrived at for each ascribed code. The agreement between the clinicians’ COD and that perceived by the participants was compared using Cohen’s Kappa coefficient. The Kappa value was categorised as poor if Kappa was ≤0.20, Fair if it was between 0.21-0.40, Moderate between 0.41-0.60, Good between 0.61-0.80 and Very Good if ≥0.80.

## Results

**Fig. 1:**
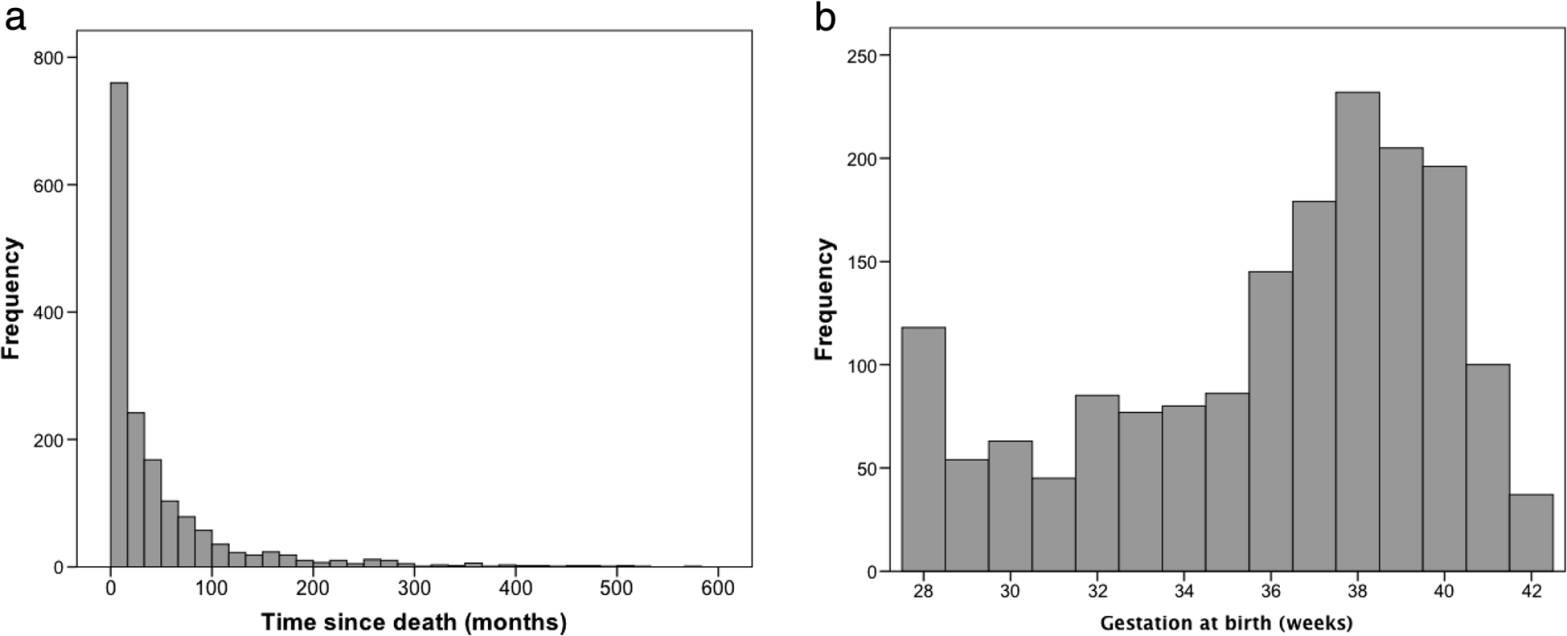
**a** Median duration of time since stillbirth **b** Median gestational age (weeks) at the time of stillbirth

In total, 1714 women who had experienced a stillbirth >3 weeks prior to enrollment completed the survey. Median duration of time since the stillbirth was 19.0 months (0.75-570.0 months; Fig. 1a). Demographics of the sample, including the participant’s country of residence, are shown in Table 1. The median age of women at the time of stillbirth was 30.0 years (18–47 years). The majority (98.6 %) of respondents were from high-income countries with 1.4 % coming from 15 different low or middle-income countries. The median gestation at the time of the stillbirth was 37 weeks (range 28–42 weeks; Fig. 1b), and 50.5 % of the babies were male.

**Table 1:** Demographic information

Median maternal age (years) (*n* = 1671)	30.0 years (range 18–47 years; IQR = 8)
Parity (*n* = 1702)	
Nulliparous	931 (54.7 %)
Gravida 1–4	754 (44.3)
Grand-Multigravida 5+	17 (1.0 %)
Country (*n* = 1696)	
USA	1208 (71.2 %)
UK	230 (13.6 %)
Australia	95 (5.6 %)
Canada	94 (5.5 %)
Ireland	15 (0.9 %)
New Zealand	10 (0.6 %)
Other*	44 (2.6 %)
Maternal Education (*n* = 1629)	
High school or less	483 (29.7 %)
Associate degree	208 (12.8 %)
Technical/trade school	122 (7.5 %)
Bachelor’s degree	490 (30.1 %)
Master’s degree	272 (16.7 %)
Doctorate degree	54 (3.2 %)

*IQR* Interquartile range

*Other (countries with <10 respondents) were Argentina *n* = 2, Belgium *n* = 2, Brazil *n* = 2, Cameroon *n* = 1, Dominican Republic *n* = 1, Ecuador *n* = 1, Fiji *n* = 1, Germany *n* = 5, Gibraltar *n* = 1, Greece *n* = 1, Guam *n* = 1, India *n* = 1, Israel *n* = 1, Japan *n* = 4, Malaysia *n* = 1, Pakistan *n* = 1, Peru *n* = 1, Philippines *n* = 2, Puerto Rico *n* = 1, Saudi Arabia *n* = 1, Singapore *n* = 1, South Africa *n* = 4, Spain *n* = 1, Switzerland *n* = 1, Netherlands *n* = 2, Trinidad & Tobago *n* = 2, United Arab Emirates *n* = 1, Vietnam = 1

### Maternal perception of fetal movements (FM)

Participants’ response to the question *“Once you were aware of your baby’s usual pattern of movements was there any time that your baby’s movements were unusual?”* is shown in Table 2. While 28.0 % reported “no change in fetal movements”, 30.5 % reported significantly less fetal movement and 8.5 % reported significantly more movement.

**Table 2:** Frequency of unusual fetal movement

	N (%)
	*N* = 1,714
No change in fetal movement	480 (28.0 %)
A little bit less movement	273 (15.9 %)
Significantly less movement	522 (30.5 %)
A little bit more movement	136 (7.9 %)
Significantly more movement	146 (8.5 %)
Don’t remember	103 (6.0 %)
Missing	54 (3.2 %)

Response to question “Once you were aware of your baby’s usual pattern of movements was there any time that your baby’s movements were unusual?”

In total, 1,077 participants reported what they did in response to the change in their baby’s movement. Table 3 illustrates the behaviour of women in response to a decrease or an increase in perception of fetal movements. It is noteworthy that significantly more women who reported increased movements, compared to those who reported reduced movements, did not worry about it (13.8 % vs. 6.4 %, *p* = 0.001) and fewer (60.7 % vs. 76.1 %, *p* < 0.001) sought professional advice from a healthcare provider. Furthermore, fewer women who reported increased movements, compared to those who reported reduced movements, were either admitted or had some type of monitoring (22.5 % vs. 32.6 %, *p* = 0.002).

**Table 3:** Response to fetal behavioural change

	Reduced movement	Increased movement
	N (%)	N (%)
	*N* = 795	*N* = 282
Did not worry	51 (6.4 %)	39 (13.8 %)
Mentioned to family and friends but did not worry further	105 (13.2 %)	59 (20.9 %)
Mentioned to healthcare provider and was reassured	244 (30.7 %)	76 (27.0 %)
Mentioned to healthcare provider and was told to monitor at home for symptoms and call back if still concerned	65 (8.1 %)	21 (7.4 %)
Mentioned to healthcare provider and had general evaluation (fetal heart rate, cervical status etc.)	59 (7.4 %)	16 (5.6 %)
Mentioned to healthcare provider and had ultrasound, biophysical profile, non-stress test, or similar (outpatient)	80 (10.1 %)	23 (8.1 %)
Mentioned to healthcare provider and was admitted for testing/monitoring	45 (5.7 %)	12 (4.5 %)
Went to emergency room or labour and delivery and was admitted	75 (9.4 %)	13 (4.6 %)
Went to emergency room or labour and delivery and was sent home	37 (4.7 %)	10 (3.5 %)
Did not provide response to question	34 (4.3 %)	13 (4.6 %)

Response to question “If you answered yes to the previous question *[baby’s movements being unusual]*, which of the following best describes your experience?

Of note, 146 (8.5 %) participants reported significantly increased fetal movements but this figure doesn’t capture the true frequency, as some women reported there was reduced movements, but commented that there was a period of increased movements which occurred prior to death. The increase was frequently described as much more “active” or “aggressive” than usual e.g.:

*“only decreased the week before birth. The day before he died he was especially busy and moving like crazy”*

*“overall movement was the same except for the last 24 hours with a big spike in movement during the day and then nothing by evening”*

*“he moved almost violently”*

*“moved like crazy then nothing”*

*“she was a little more active in the last two weeks and her pattern was slightly off. Not enough that I was concerned. I thought it was a healthy sign”*

*“The week before my baby passed, I recall she was VERY active one night when I was trying to fall asleep, so much that I actually got up out of bed for a while because her movements were keeping me awake”*

Of the four major countries represented in the data (United States, United Kingdom, Australia, and Canada), the reported frequency of unusual fetal movements was remarkably similar. Maternal response to the change in fetal movement was also similar for the United States, United Kingdom, and Australia while Canadians were less likely to seek professional advice about unusual movements (39.2 % compared to 50.6 %, 55.4 %, and 55.9 % for the United States, United Kingdom, and Australia respectively, *p* = 0.005).

### Gut instinct that something was wrong

Overall, 1,650 participants responded to the question “*During this pregnancy, did you ever have a "gut instinct" that something was wrong?”* (3.7 % did not respond). In total, 1,122 (65.5 %) responded yes to this question. Of these, 521 (46.4 %) were multiparous and 601 (53.6 %) were nulliparous. A gut instinct that something was wrong was reported by 73.4 % of women who had a stillbirth in the 6 months prior to completion of the survey. This proportion then significantly decreased to 63.6 % at 6–11.9 months post-stillbirth (*p* = 0.002) and remained remarkably stable thereafter (63.1 % at 12–17.9 months, 61.7 % at 18–23.9 months, and 63.6 % at 24 months and longer). Participants were given an opportunity to provide further comment on this response. Recollections included reports of this gut instinct some beginning early in the pregnancy, as these representative participant quotes attest:

- *“I can't explain, remember telling my partner that I had a feeling that something was going to go wrong”.*
- *“I just constantly worried something wasn't right”*
- *“Felt uneasy during entire pregnancy”.*
- *“Two days prior to my son passing, I had a routine OB visit and ultrasound. I was told everything looked great but I begged my doctor to do a C-section that day. I had an overwhelming feeling that I needed to get my son out that day. I was told that I was just being anxious”.*

### Perception of time of death

We asked participants for their perception of time of death. They were offered the following options: “In the morning 6 am-12noon”, “In the afternoon 12noon-6 pm”, “In the evening 6 pm-10 pm”, “During the night 10 pm-6 am”, “During a day-time nap” and “I’m not sure”. There were 79 missing responses and 401 who answered they were “not sure”. Together these accounted for 28.0 % of participant responses. The responses are shown in Table 4. Notably, of the *n* = 1,234 women who perceived a window of time in which they believed that their baby died, 55.8 % believed that their baby died during the night (10 pm-6 am).

**Table 4:** Maternal Perception of time of death

	N (%)
	(*N* = 1,1714)
In the morning (6 am - 12 noon)	189 (11.0 %)
In the afternoon (12 noon - 6 pm)	183 (10.7 %)
In the evening (6 pm - 10 pm)	161 (9.4 %)
During the night (10 pm - 6 am)	688 (40.1 %)
During a daytime nap	13 (0.8 %)
Not sure	401 (23.4 %)
Missing	79 (4.6 %)

Response to question “What time do you believe your baby died?”

### Investigation of stillbirth and reported cause of death

Overall, 1304 participants (76.1 %) reported that an autopsy was conducted on their baby (See Additional file 1: Table S1). Only *n* = 637; (37.2 %) had a full autopsy A minority of respondents had no autopsy but only blood testing performed on the mother (*n* = 24, 1.4 %). Critically, some respondents reported that an autopsy was not performed due to cost and/or the view that the autopsy might not give a definitive answer:

- *“OB said it wasn't necessary as they usually never find a cause and insurance won't pay for”*
- *“couldn’t afford more testing”*
- *“Asked and signed for but hospital said it just wasn’t done!”*
- *“I was told an autopsy would not provide any answers and would just be an extra expense”*
- *“No, I was told that I would have to pay a minimum of $20,000 out of pocket to have an autopsy done”*
- *“Was told there was no need for a full autopsy because they hardly ever find a cause of death”*

Participants were asked two questions regarding the cause of death (COD). Firstly, “What were you told was the cause of your baby’s death?” and secondly, “What do you believe was the cause of your baby’s death?” In response to the first question, 1002 (58.5 %) reported that they were told a COD and 593 (34.6 %) were told that their healthcare provider did not know what caused the death of their baby. With regards to their belief about COD, 1228 (71.6 %) had a belief as to what caused their baby’s death and 272 (15.9 %) did not know (Additional file 2: Table S2). Overall, there was only fair agreement between the COD reported to parents and their beliefs regarding the COD (Kappa = 0.39). The reported causes of death fell into 10 broad categories namely:

- Cord issues
- “Clotting” condition
- Other placental problems
- Fetal abnormality
- Infection
- Obstetric conditions
- Multiple causes
- Other
- My care provider played a role
- I played a role

Further detail and exploration of each of these categories, with examples, is given below:

### Cord issues

Overall, 457 (26.7 %) participants reported that they were told by their care provider that a cord accident (nuchal, true knot, velamentous, body entanglement, or prolapsed cord) was the cause of their baby’s death. While 428 (25.0 %) believed a cord accident was the cause of their baby’s death, only 312 (68.3 %) of the 457 agreed with the healthcare provider that a cord accident had occurred. Thirty-two additional participants included the cord as one of the multiple reasons they listed in what they believed caused their baby’s death. Twenty-three participants who were told by their care provider that the COD was a cord issue believed instead that their baby’s death was unexplained.

### Clotting problems (Underlying thrombophilia)

Where participants used words or phrases including the word clots, Factor V Leiden, Methylenetetrahydrofolate Reductase (MTFHR), or anti phospholipid then their baby's COD was categorized as a clotting condition (underlying thrombophilia). Overall, 71 were told that this was the COD but only 32 (45.1 %) participants agreed with their healthcare provider. An additional 30 participants believed that this was the COD having been told something else.

### Other placental factors

Responses categorized as "placental factors" (*n* = 217, 12.7 %) included those who said their healthcare provider cited placental abruption, insufficiency, or a failed placenta. Overall, 129 (7.5 %) women believed that there was a placental factor. Of the 217 who were told by their healthcare provider that the COD was due to placental factors, only 86 (39.6 %) agreed that the placenta was involved, while 31 (14.3 %) believed instead that their care provider played a role.

### Fetal abnormality

Only a few (*n* = 66, 3.9 %) of the participants were told that their baby died from a fetal abnormality. Of these, 37 (56.1 %) agreed with their provider on this issue. An additional eight participants believed their baby died from a fetal anomaly, although that is not what they were told was the COD. Few details are available in the responses regarding whether or not the fetal anomaly was lethal. For example some respondents wrote “heart defect” without indicating the type of defect.

### Infection

Infection was cited as the COD by the health care provider in 48 (2.8 %) cases and 38 participants reported this as their belief of COD. Only 22 participants (45.8 %) agreed with their healthcare provider that this was the COD. The exact type of infection was not always mentioned although eight stated that they were told the infection was Group B Streptococcus.

### Obstetric condition

There were relatively few who indicated that they were told a medical obstetric reason such as pre-eclampsia, gestational diabetes, cholestasis of pregnancy, or fetal growth restriction as a COD. We merged all of these into just one category because the total sample size was small (*n* = 62; 3.6 %). While 66 believed that their baby’s COD was related to an obstetric condition, only 20 (32.3 %) agreed with their healthcare provider that their condition was the reason for the death, with 9 (14.5 %) believing instead that the placenta played a role and 11 (17.7 %) believing that their health care provider played a role.

### Multiple reasons

If the participant cited more than one reason as the COD that was reported by their healthcare provider this was coded as a multiple reason. The responses included combinations of infection, and obstetric conditions such as fetal growth restriction, hypertension, gestational diabetes, or hemorrhage (Additional file 3: Table S3).

### Care-provider played a role

In one case the healthcare provider reportedly told the parent that they (the care provider) played a role in their baby’s death. However, a number of respondents (*n* = 138, 8.1 %) indicated that they believed that their care provider played a role. Some expressed this belief quite strongly, such as “*medical negligence* or *incompetence*” or indicated that they had legal cases pending. Others were less strong but also compelling e.g. “*being sent home the day before his death when I knew something was wrong*” and “*Not being taken seriously by labour & delivery when I went in for decreased movement*.” Interestingly, most of the women who held the belief that their care provider played a role either were told by their care providers that the death was "unknown" (*n* = 37, 26.8 %), a placental problem was likely (*n* = 31, 22.5 %), or it was the result of a cord accident (*n* = 23, 16.7 %).

### Unknown

With respect to unknown COD, 593 (34.6 %) participants indicated this as the COD they had been told by their care-provider while 272 (15.9 %) believed this to be the COD. Overall, 204 (34.4 %) participants were in agreement with their care-provider that the COD was unknown. In total, 86 (14.5 %) of those who were told the reason for their baby’s death was unknown reported their own belief that a cord accident was the COD. Of concern, 37 (6.2 %) of respondents whose COD was reported to them as “unknown” by their care-provider indicated that they felt the care provider had played a role in their baby’s death and 41 (6.9 %) believed that they themselves had played a role.

### Other

Responses which indicated that healthcare providers told parents something other than the main categories (*n* = 29) were: lack of amniotic fluid (*n* = 4), asphyxia (*n* = 2), shoulder dystocia (*n* = 2), stroke (*n* = 2), uterine rupture (*n* = 2) and one case of each of the following: fetal myocardial infarction, fetal weak heart, blood in the lungs, hypoxia, maternal fever, Rhesus antibodies, tentorial tear, cephalopelvic disproportion, cerebral haemorrhage, a fall down stairs, liver rupture, meconium aspiration, fibroids, prolonged rupture of membranes, compromised blood flow, macrosomia, and “statistics”.

### I played a role

A few (*n* = 80, 4.7 %) respondents indicated that they believed that their actions, or lack of action, played a role in their baby's death. One mother’s response is given here as it is particularly poignant but quite typical of the kind of responses the participants gave;

*“I cannot say. I fear it was my negligence in not running to the doctor when I felt her movements slow down. When her movements slowed down, I noticed and mentioned this to friends but did nothing out of fear of hearing the worst. What caused her movements to slow, I will never know, but I fear she died because I did not respond to her needs.”*

In two cases the participant was told by her healthcare provider that she played a role in her baby’s death. These two quotes are included here:

*“I was told and I quote “it’s all your fault.”*”

This women in turn reported that she considered her doctor as playing a role in her baby’s death.

The other said her care provider had told her:

*“My body treats pregnancy like cancer and fought off the pregnancy.”*

Of the n = 80 participants who believed that they had played a role in their baby's death, they had mainly been told by their care providers that their baby died from unknown reasons (*n* = 41, 51.3 %), cord accident (*n* = 11, 13.8 %), or placental involvement (*n* = 9, 11.3 %).

## Discussion

This study is one of the largest international on-line surveys ever conducted with mothers who had a stillbirth after 28 weeks gestation and provides important insight into the experiences of these women including symptoms they perceived to be associated with stillbirth. The findings of this study confirm established associations with stillbirth such as reduced fetal movements (RFM) and some newer findings such as a period of increased fetal movements, a “gut feeling” that something was wrong and the time of day of stillbirth, which merit further investigation.

It is well established that RFM is associated with increased risk of poor pregnancy outcomes such as fetal growth restriction and stillbirth [9–11]. In contrast, the impact of an *increase* in fetal movement on pregnancy outcome has been rarely reported. Whilst one recent study suggests that an increase in maternal perception of fetal movements in the last two weeks of pregnancy may be protective for stillbirth [11] others have postulated that a sudden increase in vigorous movements may indicate fetal compromise with such excessive movement perhaps being due to an hypoxic - ischaemic insult [12]. A recently reported online survey conducted in Sweden found that 10 % of a population of 215 women reported an increase in fetal movements in the 48 hours before the stillbirth [13]. The fact that 16.4 % of the participants in this study reported an increase in fetal movement prior to their baby’s death adds to this finding and indicates that the nature and timing of this change as well as an appropriate care provider response to women reporting an increase in fetal movements warrants further investigation.

Our findings strongly agree with those in a recent report from the UK that highlighted the frequency with which mothers reported RFM prior to a stillbirth and that a significant proportion (57 %) felt that they were not listened to by their care providers [14]. That many participants reported that their care provider reassured them about the change in their baby’s behaviour without taking further action is also concerning. These experiences reflect two studies that found significant variation in midwives’ and obstetricians’ practice regarding the management of RFM [15, 16]. Our findings confirm that it is important for clinicians to follow standardised clinical guidelines when managing women who report with RFM [17, 18] and that it may also be important not to discount reports of increased fetal movements. There was remarkable similarity in maternal perception of change in fetal movement and her associated behaviour in the main countries represented, perhaps suggesting that the uptake of the aforementioned guidelines by clinicians has not altered maternal response to a change in fetal movement. Although not addressed specifically in this study, the subjective change in fetal activity was perceived by mothers rather than concerns initiated by specific “alarm limits” (such as 10 kicks in 12 hours) that have not been evaluated in all pregnant women (i.e. “low” as well as “high-risk” mothers) [19]. It is therefore important that pregnant women are educated not to minimise the importance of changes in fetal movement towards the end of pregnancy as “normal”, and instead to be counselled to immediately report any change in fetal behaviour to their care provider.

### Gut instinct that something was wrong

Our findings agree with other published reports [20–22] that women who have experienced a stillbirth had a gut instinct that things may be amiss with the pregnancy, sometimes well, prior to their baby’s death. There have been no investigations of this kind in women who have had a live birth and it is probable that recall and negativity biases are very likely at play with respect to this finding. However, it is particularly noteworthy that more than two thirds of the participants who answered this question answered in the affirmative, with many recalling having these feelings from the beginning of their pregnancy and mentioning this to others well before their baby’s death. We considered that this “intuition” was associated with experience of prior pregnancies and postulated therefore that it would be more frequently reported in multiparous women however, both nulliparous and multiparous participants reported these feelings in roughly equal proportions, indeed the percentage was slightly higher in the nulliparous group. We further hypothesised that participants might have processed their feelings over time and that therefore women reporting a gut instinct might slowly increase over time; however, reports of a gut instinct were highest in the women who had a stillbirth less than 6 months ago and remarkably stable beyond the initial 6 months. Further research should therefore be initiated to explore this phenomenon, a prospective study would identify the proportion of women with this “gut instinct” in pregnancies that have a successful outcome and confirm or not the importance of identifying this feeling during pregnancy. In the meantime this maternal intuition may be something for care providers to pay attention to. We therefore suggest that if pregnant women report these feelings that they should be taken seriously and that if care providers sensitively ask them if they have these kinds of feelings, it may alert both the woman and the maternity care provider to be watchful and mindful of other ominous signs such as alteration in fetal movements or slowing fetal growth.

### Time of death

There is emerging evidence that events occurring during sleep may impact pregnancy outcome. Such events include supine sleep position [23–25], sleep disordered breathing [26] and poor sleep quality [27, 28], all of which affect a large proportion of pregnant women. While 28 % of women were unable to estimate the time of day the baby died, of the remainder, deaths were perceived to occur most frequently during the night. These findings are remarkably similar to those found in the ‘Sydney stillbirth study’ [25] where a little over 50 % of the 103 participants who had a recent stillbirth considered that their baby had died overnight. This further raises the possibility that events which occur during sleep may be important in relation to stillbirth in addition to the adverse pregnancy outcomes previously reported. Even so, events that occur during sleep may impact pregnancy outcome irrespective of the time of death. For example sleep disordered breathing is independently associated with maternal hypertension [29–34], as well as with fetal growth restriction [32, 35, 36], both of which are known risk factors for stillbirth, hence these sleep related events may, in combination, [37] be associated with stillbirth even though the stillbirth itself may not have occurred during the night. Additionally the mother may not always know exactly when the death occurred.

### Cause of death

There was fair agreement between what the participants believed caused their baby’s death and what they recall being told caused their baby’s death by their care provider. Interestingly, this is better than the Kappa value between stillbirth certificates and the COD determined by expert review (K = 0.29) [38].

It is also interesting to note the number of participants who believed (*n* = 428; 25.0 %) or were told (*n* = 457; 26.7 %) that their baby’s death was due to a cord accident of some kind, i.e., true knot, nuchal cord, or cord entanglement. This was greater than that reported in large cohort studies [39, 40]. Unfortunately, the fact that we were asking bereaved mothers about cause of death meant that we could not ask more detailed clinical questions other than what the participants offered in comment. Hence details such as degree of tightness, length of cord, amount of Wharton’s jelly, were not able to be elucidated.

Nuchal cords occur in up to 30 % of uncomplicated pregnancies and they have not been found to be associated with an increased risk of stillbirth [41]. Similarly, true knots also are quite common in live births and in order for the true knot to be implicated as the COD, demonstration of constriction of the umbilical vessels or evidence thrombosis is necessary [42]. Therefore gross cord problems (nuchal cord, true knot) in and of themselves are not usually considered the antecedent COD unless there is demonstration of vascular thrombosis/avascular villi in the placenta/fetal vessels. Nevertheless, cord-related stillbirths may be underdiagnosed. For example in a small study, when placental histologic criteria were applied to a series of 62 stillbirths, 42 % met the criteria for cord accident [43]. Therefore, it may be that a higher percentage of late stillbirths are actually caused by cord issues than is currently being attributed.

In the case where the participants believed that their baby died as a result of a cord accident and they were told something else, perhaps they did not believe or understand what their care provider told them or remembered that the cord was mentioned. Further, if they were told that the COD was unknown or the attributed cause was in some way unacceptable to them, it may be that they have adopted the belief that the cord was responsible as a means of plausible explanation either to themselves or their family and friends. Perhaps the care provider chose to implicate the cord as the COD because they considered it may be more simply understood as a physical barrier to fetal blood flow.

As well as the marked increase in the number of reported cord accidents participants also reported all COD, as told to them by their care provider, at frequencies significantly different from most perinatal mortality reports from high-income countries (e.g. [44, 45]). There was substantially less infection, and obstetric condition causes than would be expected. This may reflect exclusion of stillbirths <28 weeks gestation which are more likely to have signs of infection [46].

Conventional autopsy is considered the diagnostic gold standard, because it can confirm or augment antemortem findings, fulfil the need for parental information, assist with future planning as well as provide answers about what had happened [47]. A full autopsy can provide a diagnosis, change the antemortem clinical diagnosis, or reveal additional findings in up to 58 % of cases [47]. However, the rates of perinatal autopsy have steadily fallen worldwide, for example in the UK between 2000 and 2007, consent for perinatal autopsy declined from 55 % to 45 %, with parental objection as the main cause for conventional autopsy not being done [48]. The frequency of full autopsy in our study is comparable with this and other studies [49, 50]. It may be that in the absence of autopsy, as was the case in half of our sample, the COD as told by the care provider may be considered as unreliable as parental belief about what caused their baby’s death.

Of concern is the number of participants (*n* = 138; 8.1 %) who believed that their care provider played a role in their baby’s death. Although we acknowledge that there may be justification for some parents to blame their care provider, that so many reported that their care provider played a role in their baby’s death could reflect that maternal concerns in the antenatal or intrapartum period were not addressed [14] or indicate a lack of thorough debriefing and counselling after the stillbirth. Alternatively, it is even possible that a care-giver apology may have been warranted [51].

It is quite heartbreaking that almost five percent of the participants (*n* = 80) considered they played a role in their baby’s death especially since many reported highly unlikely and biologically implausible reasons for the stillbirth. This may reflect the high-levels of guilt often perceived by mothers after stillbirth [52].

Overall, whilst there was fair agreement (Kappa) between what the participants said they were told and what they believed was the COD it would seem likely that many of the participants either did not believe what they were told, believed that they were told something that they actually were not, or chose to believe something else. Our findings suggest that care providers delivering COD information should perhaps explore with parents what they believe was the COD in order to provide reassurance and a thorough counselling/debriefing following their stillbirth.

### Limitations of the findings

The retrospective nature of this study, participant perception, recall and negativity biases and self-selection of participants are all limitations to this study which should be taken into consideration when interpreting the results. Furthermore, women had to have access to the internet and therefore many have access to more information about factors that are believed to be associated with stillbirths e.g. reduced fetal movements [53]. Therefore, future studies to further explore our findings should ideally either be prospective or controlled.

### Strengths

In spite of the above limitations this study also has a number of strengths. Data were collected from a large number of women from across the globe, including low and middle-income countries. The surveys were completed anonymously and this may have encouraged a degree of honesty that may be greater than in a face-to-face interview, particularly demonstrated by the number of women who indicated that they believed they played a role in their baby’s death.

### Generalizability of results

The study population was mainly well-educated women living in high-income countries who were able to use English and with access to the internet. Therefore, these results may not be generalizable to other populations. As the COD of late stillbirths are very different in low and middle-income countries these results may not be generalizable to these settings. Further studies are needed in these high-burden regions (Additional file 4).

## Conclusions

Whilst it is clear that more research is needed to answer questions raised by this current study it is also apparent that several areas, particularly maternal perceptions of fetal movements and a premonition that all was not well, should be more carefully considered by maternal care providers than they currently are. Further, it is important that parents are properly counselled as to the COD so they can appreciate the cause(s) of stillbirth and care in a subsequent pregnancy can be adequately prepared.

### Recommendations

Further research is required particularly aimed at investigating the role of perceived change in fetal behaviour, especially increase in fetal movements. Another area that warrants investigation is the role of maternal sleep. It is also important to further explore how prevalent a premonition of adverse outcome might be amongst all pregnant women as if it is more common in the stillborn population this may assist care providers to carefully monitor pregnancies in which the mother reports this phenomenon. Once a stillbirth occurs it is important for the care-provider to work with the parents to correctly identify the COD by ensuring appropriate investigation and complete counselling, as assignment of a probable COD is important both to help individuals in appropriate planning of a subsequent pregnancy and to develop interventions for stillbirth prevention worldwide.

## Additional files

Additional file 1: Table 1. Components of autopsy. (DOC 32 kb). Additional file 2: Table 2. Told Cause of Death vs. Believed Cause of Death Comparison Table. (DOC 53 kb) Additional file 3: Table 3. Results of the 43 responses coded as “Multiple Reasons” for the healthcare provider reported COD. (DOC 29 kb) Additional file 4:
**Stillbirth 'cohort' survey**. (PDF 266 kb)

## Abbreviations

COD: Cause of death
RFM: Reduced fetal movements
STARS: Study of Trends and Associated Risks for Stillbirth

## References

[1] Macfarlane A, Dattani N, Mohangoo A, Zeitlin J (2013). What can the UK learn from international comparisons of routinely collected perinatal data? UK perspectives on the Euro-Peristat project. Lancet.

[2] Flenady V, Middleton P, Smith GC, Duke W, Erwich JJ, Khong TY (2011). Stillbirths: the way forward in high-income countries. Lancet.

[3] Goldenberg RL, McClure EM, Bhutta ZA, Belizan JM, Reddy UM, Rubens CE (2011). Stillbirths: the vision for 2020. Lancet.

[4] Flenady V, Koopmans L, Middleton P, Frøen JF, Smith GC, Gibbons K (2011). Major risk factors for stillbirth in high-income countries: a systematic review and meta-analysis. Lancet.

[5] Reddy UM, Laughon SK, Sun L, Troendle J, Willinger M, Zhang J (2010). Prepregnancy risk factors for antepartum stillbirth in the United States. Obstet Gynecol.

[6] The Stillbirth Collaborative Research Network Writing Group (2011). Association Between Stillbirth and Risk Factors Known at Pregnancy Confirmation. JAMA.

[7] Mitchell EA (2012). Emerging ideas to better understand and prevent stillbirths. BMC Pregnancy Childbirth.

[8] Dillman DA, Tortora RD, & Bowker D. Principles for Constructing Web Surveys. SESRC Technical Report 98–50, 1998 Pullman, Washington. Accessed 8th May 2014 http://www.sesrc.wsu.edu/dillman/papers/1998/principlesforconstructingwebsurveys.pdf

[9] Holm Tveit JV, Saastad E, Stray-Pedersen B, Bordahl PE, Froen JF (2009). Maternal characteristics and pregnancy outcomes in women presenting with decreased fetal movements in late pregnancy. Acta Obstet Gynecol Scand.

[10] O'Sullivan O, Stephen G, Martindale E, Heazell AE (2009). Predicting poor perinatal outcome in women who present with decreased fetal movements. J Obstet Gynaecol.

[11] Stacey T, Thompson JM, Mitchell EA, Ekeroma A, Zuccollo J, McCowan LM (2011). Maternal perception of fetal activity and late stillbirth risk: findings from the Auckland Stillbirth Study. Birth.

[12] Sadovsky E, Polishuk WZ (1977). Fetal movements in utero: nature, assessment, prognostic value, timing of delivery. Obstet Gynecol.

[13] Linde A, Pettersson K, Rådestad I (2015). Women's Experiences of Fetal Movements before the Confirmation of Fetal Death--Contractions Misinterpreted as Fetal Movement. Birth.

[14] Redshaw M, Rowe R, Henderson J (2014). Listening to Parents after stillbirth or the death of their baby after birth.

[15] Heazell AE, Green M, Wright C, Flenady V, Frøen JF (2008). Midwives' and obstetricians' knowledge and management of women presenting with decreased fetal movements. Acta Obstet Gynecol Scand.

[16] Flenady V, MacPhail J, Gardener G, Chadha Y, Mahomed K, Heazell A (2009). Detection and management of decreased fetal movements in Australia and New Zealand: a survey of obstetric practice. Aust N Z J Obstet Gynaecol.

[17] Preston S, Mahomed K, Chadha Y, Flenady V, Gardener G, MacPhail J (2010). Clinical practice guideline for the management of women who report decreased fetal movements.

[18] RCOG. 2011. Green-top Guideline 57: Reduced fetal movements. London: Royal College of Obstetricians and Gynaecologists. Available from http://www.rcog.org.uk (Accessed 31 July 2015).

[19] Heazell AE, Frøen JF (2008). Methods of fetal movement counting and the detection of fetal compromise. J Obstet Gynaecol.

[20] Trulsson O, Rådestad I (2004). The silent child--mothers' experiences before, during, and after stillbirth. Birth.

[21] Erlandsson K, Lindgren H, Davidsson-Bremborg A, Rådestad I (2012). Women's premonitions prior to the death of their baby in utero and how they deal with the feeling that their baby may be unwell. Acta Obstet Gynecol Scand.

[22] Malm M-C, Lindgren H, Rådestad I: Losing contact with one’s unborn baby – mothers’ experiences prior to receiving news that their baby has died in utero. Omega (Westport) 2010*–*2011, 62:353–367. 10.2190/om.62.4.c 21661539

[23] Stacey T, Thompson JM, Mitchell EA, Ekeroma AJ, Zuccollo JM, McCowan LM (2011). Association between maternal sleep practices and risk of late stillbirth: a case–control study. BMJ.

[24] Owusu T, Anderson FJ, Coleman J, Oppong S, Seffah JD, Obed S (2013). Association of maternal sleep practices with pre-eclampsia, low birth weight, and stillbirth among Ghanaian women. Int J Gynecol Obstetr.

[25] Gordon A, Raynes-Greenow C, Bond D, Morris J, Rawlinson W, Jeffery H (2015). Sleep position, fetal growth restriction, and late-pregnancy stillbirth. Obstet Gynecol.

[26] Pamidi S, Pinto LM, Marc I, Benedetti A, Schwartzman K, Kimoff RJ (2014). Maternal sleep-disordered breathing and adverse pregnancy outcomes: a systematic review and metaanalysis. Am J Obstet Gynecol.

[27] Lee KA, Gay CL (2004). Sleep in late pregnancy predicts length of labor and type of delivery. Am J Obstet Gynecol.

[28] Okun ML, Schetter CD, Glynn LM (2011). Poor sleep quality is associated with preterm birth. Sleep.

[29] Perez-Chada D, Videla AJ, O’Flaherty ME, Majul C, Catalini AM, Caballer CA (2007). Snoring, witnessed sleep apnoeas, and pregnancy-induced hypertension. Acta Obstet Gynecol Scand.

[30] O'Brien LM, Bullough AS, Owusu JT, Tremblay KA, Brincat CA, Chames MC (2012). Pregnancy-Onset Habitual Snoring, Gestational Hypertension, and Pre-eclampsia: Prospective Cohort Study. Am J Obstet Gynecol.

[31] Louis JM, Mogos MF, Salemi JL, Redline S, Salihu HM (2014). Obstructive sleep apnea and severe maternal-infant morbidity/mortality in the United States, 1998–2009. Sleep.

[32] Franklin KA, Holmgren PA, Jonsson F, Poromaa N, Stenlund H, Svanborg E (2000). Snoring, pregnancy-induced hypertension, and growth retardation of the fetus. Chest.

[33] Bourjeily G, Raker CA, Chalhoub M, Miller MA (2010). Pregnancy and fetal outcomes of symptoms of sleep-disordered breathing. Eur Respir J.

[34] Louis JM, Auckley D, Sokol RJ, Mercer BM (2010). Maternal and neonatal morbidities associated with obstructive sleep apnea complicating pregnancy. Am J Obstet Gynecol.

[35] O’Brien LM, Bullough AS, Owusu JT, Tremblay KA, Brincat CA, Chames MC (2013). Habitual snoring during pregnancy and delivery outcomes: prospective cohort study. Sleep.

[36] Fung AM, Wilson DL, Lappas M, Howard M, Barnes M, O'Donoghue F (2013). Effects of maternal obstructive sleep apnoea on fetal growth: a prospective cohort study. PLoS One.

[37] Warland J, Mitchell EA (2014). A triple risk model for unexplained late stillbirth. BMC Pregnancy Childbirth.

[38] Cockerill R, Whitworth MK, Heazell AE (2012). Do medical certificates of stillbirth provide accurate and useful information regarding the cause of death?. Paediatr Perinat Epidemiol.

[39] Gardosi J, Kady SM, McGeown P, Francis A, Tonks A (2005). Classification of stillbirth by relevant condition at death (ReCoDe): population based cohort study. BMJ.

[40] Froen JF, Pinar H, Flenady V, Bahrin S, Charles A, Chauke L (2009). Causes of death and associated conditions (Codac): a utilitarian approach to the classification of perinatal deaths. BMC Pregnancy Childbirth.

[41] Carey JC, Rayburn WF (2000). Nuchal cord encirclements and risk of stillbirth. Int J Gynaecol Obstet.

[42] Ryan WD, Trivedi NA, Benirschke K, Lacoursiere DY, Parast M (2012). Placental histologic criteria for diagnosis of cord accident: sensitivity and specificity. Pediatr Dev Pathol.

[43] Parast MM, Crum CP, Boyd TK (2008). Placental histologic criteria for umbilical blood flow restriction in unexplained stillbirth. Hum Pathol.

[44] Centre for Maternal and Child Enquiries (CMACE) (2011). Perinatal Mortality 2009: United Kingdom.

[45] Li Z, Zeki R, Hilder L, Sullivan E, Hilder L, Sullivan EA (2011). Australia’s mothers and babies. Perinatal statistics 2013 series no. 28. Cat. no. PER 59.

[46] Pinar H, Goldenberg RL, Koch MA, Heim-Hall J, Hawkins HK, Shehata B (2014). Placental Findings in Singleton Stillbirths. Obstet Gynecol.

[47] Burton JL, Underwood J (2007). Clinical, educational, and epidemiological value of autopsy. Lancet.

[48] Khong TY (1996). A review of perinatal autopsy rates worldwide, 1960s to 1990s. Paediatr Perinat Epidemiol.

[49] Bishop KL, Dupuis C, Nanton P, Clarke K, Bolt C, Chin-See C (2013). Perinatal autopsy rates at the University Hospital of the West Indies: 2002–2008. West Indian Med J.

[50] Tan GC, Hayati AR, Khong TY (2010). Low Perinatal Autopsy Rate in Malaysia: Time for a Change. Pediatr Dev Pathol.

[51] Ho B, Liu E (2011). Does sorry work? The impact of apology laws on medical malpractice. J Risk Uncertainty.

[52] Barr P (2004). Guilt- and shame-proneness and the grief of perinatal bereavement. Psychol Psychother.

[53] McArdle A, Flenady V, Toohill J, Gamble J, Creedy D (2015). How pregnant women learn about foetal movements: sources and preferences for information. Women Birth.

