# Supplementary material for: An international internet survey of the experiences of 1,714 mothers with a late stillbirth: the STARS cohort study
Source: BMC Pregnancy Childbirth. 2015 Aug 15;15:172. doi: 10.1186/s12884-015-0602-4 (PMC4537542; doi:10.1186/s12884-015-0602-4)
Supplement: Additional file 1: Table 1. — Components of autopsy. (DOC 32 kb). [file 12884_2015_602_MOESM1_ESM.doc]

**Supplementary Table 1: Components of autopsy**

|  | **N (%)**  **(N=1,714)** |
| --- | --- |
| Full autopsy | 637 (37.2%) |
| Placenta | 874 (51.0%) |
| Cord | 663 (38.7%) |
| Non-invasive exam | 439 (25.6%) |
| Blood test on baby | 413 (24.1%) |
| Blood test on mother | 696 (40.6%) |
| Chromosome/genetic test | 360 (21.0%) |
| Tissue samples | 289 (16.9%) |
| Imaging | 65 (3.8%) |
| No autopsy | 336 (19.6%) |
| Missing | 74 (4.3%) |
