# Supplementary material for: An international internet survey of the experiences of 1,714 mothers with a late stillbirth: the STARS cohort study
Source: BMC Pregnancy Childbirth. 2015 Aug 15;15:172. doi: 10.1186/s12884-015-0602-4 (PMC4537542; doi:10.1186/s12884-015-0602-4)
Supplement: Additional file 2: Table 2. — Told Cause of Death vs. Believed Cause of Death Comparison Table. (DOC 53 kb) [file 12884_2015_602_MOESM2_ESM.doc]

**Supplementary Table 2: Told Cause of Death vs. Believed Cause of Death Comparison Table**

|  | **Parental Belief of COD** | | | | | | | | | | | | | Total |
| --- | --- | --- | --- | --- | --- | --- | --- | --- | --- | --- | --- | --- | --- | --- |
| **Provider told COD** |  | Cord Issue | Placental Factor | Fetal Abnormality | Infection | Clotting Condition | Obstetric Condition | Care Provider | Self | Multiple Reasons | Other | Don’t Know | Missing |  |
|  | Cord Issue | 312 | 3 | 0 | 0 | 7 | 3 | 23 | 11 | 40 | 9 | 23 | 26 | 457 |
|  | Placental Factor | 6 | 86 | 2 | 0 | 4 | 7 | 31 | 9 | 22 | 11 | 19 | 20 | 217 |
|  | Fetal Abnormality | 4 | 2 | 37 | 0 | 1 | 0 | 5 | 0 | 4 | 5 | 3 | 5 | 66 |
|  | Infection | 1 | 0 | 1 | 22 | 1 | 0 | 6 | 4 | 7 | 1 | 2 | 3 | 48 |
|  | Clotting Condition | 3 | 7 | 0 | 0 | 32 | 0 | 7 | 6 | 9 | 1 | 3 | 3 | 71 |
|  | Obstetric Condition | 0 | 9 | 0 | 0 | 1 | 20 | 11 | 4 | 6 | 2 | 6 | 3 | 62 |
|  | Care Provider | 0 | 0 | 0 | 0 | 0 | 0 | 1 | 0 | 0 | 0 | 0 | 0 | 1 |
|  | Self | 0 | 0 | 0 | 0 | 0 | 0 | 1 | 0 | 0 | 0 | 1 | 0 | 2 |
|  | Multiple Reasons | 5 | 5 | 0 | 4 | 1 | 2 | 5 | 3 | 13 | 1 | 5 | 3 | 47 |
|  | Other | 2 | 1 | 0 | 2 | 1 | 0 | 9 | 1 | 2 | 8 | 3 | 2 | 31 |
|  | Don’t Know | 86 | 16 | 5 | 8 | 12 | 31 | 37 | 41 | 56 | 41 | 204 | 56 | 593 |
|  | Missing | 9 | 0 | 0 | 2 | 2 | 3 | 2 | 1 | 3 | 1 | 3 | 93 | 119 |
| Total |  | 428 | 129 | 45 | 38 | 62 | 66 | 138 | 80 | 162 | 80 | 272 | 214 | 1714 |
