# Supplementary material for: An international internet survey of the experiences of 1,714 mothers with a late stillbirth: the STARS cohort study
Source: BMC Pregnancy Childbirth. 2015 Aug 15;15:172. doi: 10.1186/s12884-015-0602-4 (PMC4537542; doi:10.1186/s12884-015-0602-4)
Supplement: Additional file 3: Table 3. — Results of the 43 responses coded as “Multiple Reasons” for the healthcare provider reported COD. (DOC 29 kb) [file 12884_2015_602_MOESM3_ESM.doc]

**Supplementary Table 3: Results of the 43 responses coded as “Multiple Reasons” for the healthcare provider reported COD**

|  | **N** |
| --- | --- |
| Cord or placental issue | 32 |
| Clots | 14 |
| Infection | 10 |
| Obstetric condition (n=14)  Fetal Growth Restriction  Hypertension  Gestational diabetes  Haemorrhage  Polyhydramnios  No amniotic fluid | 6  2  2  2  1  1 |
| Fetal Anomaly | 1 |
| Post-dates | 1 |
