# Supplementary material for: An international internet survey of the experiences of 1,714 mothers with a late stillbirth: the STARS cohort study
Source: BMC Pregnancy Childbirth. 2015 Aug 15;15:172. doi: 10.1186/s12884-015-0602-4 (PMC4537542; doi:10.1186/s12884-015-0602-4)
Supplement: Additional file 4: — Stillbirth 'cohort' survey. (PDF 266 kb) [file 12884_2015_602_MOESM4_ESM.pdf]

New Survey

If you meet the following qualifications, please continue.

1. I confirm that I....

*** had a single stillborn baby and,**
*** had this baby at 28 weeks gestation or more and,**
*** was at least 18 years old when this baby was born and**
*** am able to read, write and understand English and**
*** can clearly recall very specific details about my pregnancy and delivery**

◯ Yes, I am qualified to participate in this study.

◯ No, I do not meet all of the qualification criteria.

New Survey

Thank you for your interest in participating in the STARS Study. Your participation will be valuable in helping to learn more about the causes of stillbirth.

These first few questions are to gather some general information about you at the time of this pregnancy.

2. What is today's date?

	MM		DD		YYYY
Please enter today's date.		/		/	

3. What is your date of birth?

	MM		DD		YYYY
Please enter date you were born.		/		/	

4. How tall are you? (Please indicate feet/inches or meters)

	Feet	Inches	Meters
Please enter your height in either feet/inches or centimeters			

5. How would you describe your race/ethnicity?

- ○ Caucasian
- ○ African American/Black
- ○ Alaska Native
- ○ Native American/Canadian
- ○ Asian
- ○ Latino/Hispanic
- ○ Pacific Islander
- ○ Middle Eastern
- ○ Australian Aboriginal
- ○ Mixed race

Other (please specify)

6. What is your highest level of education completed?

- Some high school
- High School Diploma
- Associates Degree
- Technical/Trade School Degree
- Bachelor's Degree
- Master's Degree
- Doctorate Degree
- Post-doctoral education

Other (please specify)

7. How would you describe your employment status during this pregnancy?

- Unemployed
- Stay at home Mom.
- Worked from home part time.
- Worked from home full time.
- Worked outside the home part time.
- Worked outside the home full time.
- Other (please specify)

8. If you worked outside of the home during this pregnancy, was it shift work?

- No
- Yes, day shift
- Yes, evening shift
- Yes, night shift
- Yes, rotating shifts
- I do not work outside of the home.

9. If you live in the United States, were you covered by private health insurance for this pregnancy?

- ◯ Yes
- ◯ No

If you live outside the United States please describe any financial coverage for this pregnancy.

10. What was your combined household annual income before taxes at the time of this pregnancy?

- ◯ Less than $25,000
- ◯ $25,000 - $50.000
- ◯ $50,000 - $100,000
- ◯ $100,000 - $200,000
- ◯ More than $200,000
- ◯ Prefer not to answer

For those outside the United States - please enter your salary in your local currency.

11. What best describes your relationship with this baby's father during this pregnancy?

- ◯ Married
- ◯ Engaged
- ◯ Partners
- ◯ Long term relationship
- ◯ Dating
- ◯ Acquainted
- ◯ No relationship

Other (please specify)

12. How old was the father of your child at the time of this pregnancy? (If unknown, do not answer)

13. How would the father of this child describe his race/ethnicity?

- ◯ Caucasian
- ◯ African American/Black
- ◯ Alaskan Native or American Indian
- ◯ Asian
- ◯ Latino/Hispanic
- ◯ Pacific Islander
- ◯ Middle Eastern
- ◯ Australian Aboriginal
- ◯ Mixed race
- ◯ Unknown
- ◯ Other (please specify)

14. When was this baby born?

	MM		DD		YYYY
Please enter date		/		/	

15. What is this baby's gender?

- ◯ Male
- ◯ Female
- ◯ Uncertain

New Survey

Please answer ONLY ONE of the following three questions depending on whether you prefer to answer in pounds and ounces, kilos or grams.

16. Please provide your baby's birth weight in pounds/ounces.

	Poundsunces	O
Enter birth weight in pounds/ounces.		

17. Please provide your baby's birth weight in kilos.

	Kilos
Baby's weight in kilos	

18. Please enter your baby's birth weight in grams.

19. Where did you deliver this baby?

- Hospital
- Out of hospital birth center
- At Home

Other (please specify)

20. How many weeks pregnant were you when this baby was born?

- 28
- 29
- 30
- 31
- 32
- 33
- 34
- 35
- 36
- 37
- 38
- 39
- 40
- 41
- 42 or more
- Uncertain, please explailn

21. Which of the following describes your main healthcare provider during this pregnancy?

- Obstetrician
- Family Physician
- Midwife - CNM (United States)
- Midwife - CPM (United States)
- Midwife (International Provider)
- Maternal/Fetal Medicine Specialist
- Perinatologist
- Did not have prenatal care during this pregnancy

Other (please specify)

22. Did you change healthcare providers at any time during this pregnancy?

- No
- Yes, (please explain)

23. What was your weight before this pregnancy? (Please specify either pounds/ounces or kilos). Leave blank if you do not remember.

	Pounds	Kilos
Enter closest pre-pregnancy weight		

24. How much weight did you gain during this pregnancy? (Please specify pounds/ounces or kilograms) Leave blank if you do not remember.

	Pounds	Ounces	Kilos
Enter weight gained during this pregnancy.			

25. How many weeks pregnant were you when you first visited a healthcare provider about your pregnancy?

	Weeks
Select Number of Weeks Pregnant	

26. Were you considered 'high risk' for this pregnancy?

- ○ No
- ○ Yes, due to a medical condition diagnosed before pregnancy
- ○ Yes, due to a medical condition diagnosed during pregnancy
- ○ Yes, due to a previous pregnancy complication
- ○ Yes, due to a previous pregnancy loss
- ○ I don't know

If yes, please describe

27. Did you have any medical conditions prior to this pregnancy?

- ○ No
- ○ Yes
- ○ I don't remember

If yes, please describe

28. Were you diagnosed with or treated for any medical conditions during this pregnancy?

- ○ No
- ○ Yes
- ○ I don't remember.

If yes, please describe

29. Did you use fertility treatments to become pregnant with this baby?

- ◯ No
- ◯ Yes

If yes, please describe:

30. How many pregnancies did you have BEFORE this pregnancy?

- ◯ 0
- ◯ 1
- ◯ 2
- ◯ 3
- ◯ 4
- ◯ 5
- ◯ 6
- ◯ 7
- ◯ 8
- ◯ 9
- ◯ 10
- ◯ More than 10

31. How many live children have you given birth to BEFORE this pregnancy?

- ◯ 0
- ◯ 1
- ◯ 2
- ◯ 3
- ◯ 4
- ◯ 5
- ◯ 6
- ◯ 7
- ◯ 8
- ◯ 9
- ◯ 10
- ◯ More than 10

32. BEFORE this pregnancy, have you had any pregnancies end in the first trimester (first 12 weeks)?

- ◯ No
- ◯ Yes

If yes, how many?

33. BEFORE this pregnancy, did you have any pregnancy losses after 12 weeks?

- ◯ No
- ◯ Yes

If yes, how many?

34. Did this baby have a congenital abnormality (genetic condition, physical abnormality, etc.)?

- ◯ Yes
- ◯ No
- ◯ I don't know

If yes, please describe

35. How would you describe your activity level BEFORE this pregnancy?

◯ INACTIVE - rarely walk anywhere, have a sedentary job that involves sitting for long periods, and generally you don’t do any specific exercise during an average day.

◯ LIGHT ACTIVITY - You might have a light walk to and from work, or you may have a job that means you spend periods of time on your feet or walking around. You may also occasionally visit a gym or take part in exercise classes.

◯ MODERATE ACTIVITY - you exercise regularly for 30 minutes or more, up to 5 x/week, you will also probably have a hobby that involves moderate activity, or maybe you take a dog for a long walk several times a week .

◯ ACTIVE - regular exercise for 30 minutes or more, 5 x/week or more. You are also likely to walk often during your day and have a hobby that includes involves a high level of activity.

36. How would you describe your usual activity level DURING this pregnancy?

◯ INACTIVE - rarely walk anywhere, have a sedentary job that involves sitting for long periods, and generally you don’t do any specific exercise during an average day.

◯ LIGHT ACTIVITY - You might have a light walk to and from work, or you may have a job that means you spend periods of time on your feet or walking around. You may also occasionally visit a gym or take part in exercise classes.

◯ MODERATE ACTIVITY - you exercise regularly for 30 minutes or more, up to 5 x/week, you will also probably have a hobby that involves moderate activity, or maybe you take a dog for a long walk several times a week .

◯ ACTIVE - regular exercise for 30 minutes or more, 5 x/week or more. You are also likely to walk often during your day and have a hobby that includes involves a high level of activity.

◯ MEDICALLY PRESCRIBED BED REST - due to a concern with your pregnancy you were told to remain inactive on bedrest for a significant time during this pregnancy.

37. Did you have any ultrasound examinations during this pregnancy?

- ◯ No
- ◯ Yes, one.
- ◯ Yes, two.
- ◯ Yes, three or more.

38. If you answered yes to the previous question, were any of these ultrasounds a Level 2 (anatomy examination) ultrasound or done with a biophysical profile (a longer ultrasound watching for baby's breathing-like activity and movements).

- ◯ Yes
- ◯ No
- ◯ I don't know.
- ◯ Not applicable.

39. During an ultrasound, were any abnormalities or concerns identified?

- ◯ No
- ◯ Yes
- ◯ I do not know
- ◯ Not applicable

If yes, please describe

40. What was the position of the placenta noted during ultrasound?

- ◯ Anterior
- ◯ Posterior
- ◯ Fundal
- ◯ Lateral
- ◯ Previa
- ◯ Other
- ◯ I don't know

Please describe:

41. During an ultrasound examination, did your healthcare provider comment on any other characteristics of your baby's umbilical cord before birth? (Choose all that apply)

- [ ] I don't remember
- [ ] No
- [ ] Yes, the cord was around the baby's neck
- [ ] Yes, there was extra twisting of the cord
- [ ] Yes, there was no twisting of the cord
- [ ] Yes, abnormal insertion into the placenta
- [ ] Yes, cord was wrapped around body parts other than the neck
- [ ] Yes, a knot(s) was identified in the cord
- [ ] Yes, the umbilical cord only had two blood vessels

Other (please specify)

42. Did you experience any vaginal bleeding during this pregnancy?

- No
- Yes, a single episode before 20 weeks.
- Yes, recurrent bleeding before 20 weeks.
- Yes, single episode after 20 weeks.
- Yes, recurrent bleeding after 20 weeks.
- Yes, recurrent bleeding throughout the pregnancy.
- I don't remember.

43. Did you smoke tobacco during this pregnancy?

- Yes
- No, I have never smoked.
- No, I stopped before this pregnancy.
- No, I stopped during pregnancy.
- I tried to quit but restarted during pregnancy.
- I quit smoking but used nicotine patches.

44. If you smoked at any time during this pregnancy, how many cigarettes did you smoke on average each day?

45. Did anyone living in your household (other than yourself) smoke during this pregnancy?

- No
- Yes

46. During this pregnancy did you work in an environment where you were exposed to second hand smoke?

- ◯ Yes
- ◯ No

47. During this pregnancy, did you use any recreational drugs (marijuana, cocaine, etc)?

- ◯ No
- ◯ Yes, once
- ◯ Yes, occasionally
- ◯ Yes, frequently

48. During this pregnancy, did you use any of the following:

	Never	1-2 times throughout pregnancy	monthly	weekly	daily
Alcohol	◯	◯	◯	◯	◯
Prescripition drugs	◯	◯	◯	◯	◯
Over the counter drugs	◯	◯	◯	◯	◯
Vitamins	◯	◯	◯	◯	◯
Dietary supplements	◯	◯	◯	◯	◯
Herbal remedies	◯	◯	◯	◯	◯
Folk remedies	◯	◯	◯	◯	◯
Complimentary therapies	◯	◯	◯	◯	◯
Alternative therapies	◯	◯	◯	◯	◯
Naturopathic therapies	◯	◯	◯	◯	◯
Holistic therapies	◯	◯	◯	◯	◯

Please describe your answers (other than never)

49. In what range was your usual blood pressure before you were pregnant?

- ◯ Very low
- ◯ Borderline low
- ◯ Normal
- ◯ Borderline high
- ◯ Very high
- ◯ I don't know

If you know your usual blood pressure when not pregnant please provide it here

50. During this pregnancy, in what range was your blood pressure?

- ◯ Low
- ◯ Borderline low
- ◯ Normal
- ◯ Borderline high
- ◯ High

If you know your usual blood pressure during this pregnancy please provide it here

51. Did you take medication/herbal remedies for sleep during this pregnancy (either prescription or over the counter)?

- Yes
- No

If yes, please specify

52. How would you rate your overall sleep quality?

- Very good
- Fairly good
- Average
- Fairly poor
- Very poor

The next questions ask about your baby's movements.

53. During this pregnancy did your healthcare provider tell you about or ask you to keep track of your baby's movement.

- ◯ Yes
- ◯ No
- ◯ I don't remember

54. Did you keep track of your baby's movement during this pregnancy?

- ◯ Yes
- ◯ No

If yes, please describe the method you used to keep track of your baby's movements

55. How would you describe this baby's usual movements?

- ◯ Little to no movement felt
- ◯ Infrequent movements
- ◯ Average movements
- ◯ Above average movements
- ◯ Constant movement
- ◯ I don't remember

56. Once you were aware of your baby's usual pattern of movement, was there any time your baby's movements were unusual?

- ◯ No
- ◯ Yes, a little bit more
- ◯ Yes, significantly more
- ◯ Yes, a little bit less
- ◯ Yes, significantly less
- ◯ I don't remember

If yes, please explain

57. If you answered yes to the previous question, did you seek caregiver advice for this change?

- ◯ Yes
- ◯ No
- ◯ Not applicable

If yes, how many weeks pregnant were you when this occurred.

58. During the last two weeks of this pregnancy, were the STRENGTH of your baby's movements....

- ◯ Increased
- ◯ Decreased
- ◯ Stayed the same
- ◯ I don't remember

Please describe:

59. During the last two weeks of this pregnancy, did the FREQUENCY of your baby's movements....

- ◯ Increase
- ◯ Decrease
- ◯ Stay the same
- ◯ I don't remember

Please describe:

60. Did you usually feel your baby move at bedtime during this pregnancy?

- ◯ Yes
- ◯ No
- ◯ I don't remember

61. Did you feel your baby move at bedtime on the last night of this pregnancy?

- ◯ Yes
- ◯ No
- ◯ I don't remember

62. During the last two weeks of this pregnancy, did you notice any time that your baby was more vigorous than usual?

- ◯ No
- ◯ Yes, once.
- ◯ Yes, sometimes.
- ◯ Yes, often.
- ◯ I don't remember.

If yes, please describe:

63. If you noticed your baby having hiccup like movements, how long would each episode last on average?

- ◯ Less than 5 minutes
- ◯ 5-10 minutes
- ◯ More than 10 minutes
- ◯ No hiccups
- ◯ I don't remember

64. If your baby experienced hiccup like movements, how often did you notice them?

- ◯ Once or twice thought this pregnancy
- ◯ Weekly
- ◯ Daily
- ◯ More than 3 times per day
- ◯ I don't remember

Please describe:

65. If you experienced a change in your baby's behavior, which of the following best describes your experience (check all that apply)

- ☐ Did not worry about it
- ☐ Mentioned to family/friends but didn't not worry
- ☐ Mentioned to my healthcare provider and was reassured
- ☐ Mentioned to my healthcare provider and was told to monitor for symptoms at home or call again if more concerned
- ☐ Mentioned to my healthcare provider and was evaluated by checking the baby's heart beat
- ☐ Mentioned to my healthcare proficer and was evaluated with ultrasound, non-stress test, biophysical profile or similar testing.
- ☐ Mentioned to my healthcare provider and was admitted to emergency room or hospital for testing and monitoring
- ☐ Went to emergency room or labor and delivery and was sent home.
- ☐ Went to emergency room or labor and delivery and was evaluated with ultrasound and/or fetal heart rate monitoring.
- ☐ Not applicable.

66. During the last two weeks of this pregnancy, did you feel contractions, pre-labor contractions, Braxton-Hick contractions or false labor for longer than an hour at any one time?

- ◯ Yes
- ◯ No
- ◯ I don't remember.

67. If you answered yes to the previous question, which of the following describes your experience?

- ◯ Did not worry about it
- ◯ Mentioned to family/friends but did not worry
- ◯ Mentioned to my healthcare provider and was reassured
- ◯ Mentioned to my healthcare provider and was told to monitor for symptoms at home or call if more concerned.
- ◯ Mentioned to my healthcare provider and was evaluated with a general examination (baby's heart rate, amount of cervical dialation/effacement, etc)
- ◯ Mentioned to my healthcare provider and was evaluated with ultrasound, non-stress test, biophysical profile or similar testing
- ◯ Mentioned to my healthcare provider and was admitted to emergency room or hospital for testing and monitoring
- ◯ Went to the emergency room or labor & delivery and was sent home.
- ◯ Went to the emergency room or labor & delivery and was evaluated with ultrasound, fetal heart rate monitoring and/or physical exam.
- ◯ Not applicable.

68. Were you TESTED for any viral or bacterial infections during this pregnancy?

- ◯ Yes
- ◯ No
- ◯ I don't know.

If yes, please describe

69. Were you TREATED for any infections during this pregnancy?

- ◯ No
- ◯ Yes
- ◯ I don't know

If yes, please describe

70. If you were treated for an infection, were you retested to be sure the infection was gone?

- ◯ Yes
- ◯ No
- ◯ I don't know
- ◯ Not applicable

71. Were your membranes stripped or swept by your healthcare provider during this pregnancy?

- ◯ No
- ◯ Yes
- ◯ I don't know.

If yes, how long was it done before the baby was born?

72. If your membranes were stripped or swept, please tell us why this was done.

73. Did you take any antibiotics during this pregnancy?

- ◯ No
- ◯ Yes, during the first trimester
- ◯ Yes, during the second trimester
- ◯ Yes, during the third trimester
- ◯ Yes, during labor
- ◯ I don't know

If yes, what were the antibiotics given to you for?

74. If yes to the previous question, what was the name of the antibiotic you were given. Leave blank if you do not remember.

75. Were you sexually active during this pregnancy?

- ◯ No
- ◯ Yes, rarely
- ◯ Yes, occasionally
- ◯ Yes, frequently

76. Did you have more than one sexual partner during this pregnancy?

- ◯ Yes
- ◯ No

77. Was an autopsy or other post mortem examination performed? (Select all that apply.)

- [ ] No
- [ ] Yes, placenta
- [ ] Yes, umbilical cord
- [ ] Yes, non-invasive exam of baby
- [ ] Yes, blood tests on baby
- [ ] Yes, blood tests on Mom
- [ ] Yes, chromosome/genetic studies
- [ ] Yes, tissue samples
- [ ] Yes, full autopsy
- [ ] Yes, imaging (xray, MRI, CT, etc.)
- [ ] Other: (please describe)

78. Did your healthcare provider comment on the length of your baby's umbilical cord?

- ( ) No
- ( ) Yes, it was shorter than normal.
- ( ) Yes, it was normal length
- ( ) Yes, was longer than normal
- ( ) I don't remember.

79. Did your healthcare provider comment on any other characteristics of your baby's umbilical cord after delivery?

- ◯ I don't remember
- ◯ No
- ◯ Yes, the cord was around my baby's neck
- ◯ Yes, there was extra twisting of the cord
- ◯ Yes, there was no twisting of the cord
- ◯ Yes, the cord was abnormally inserted into the placenta
- ◯ Yes, the cord was wrapped around body parts other than the neck
- ◯ Yes, there was a knot(s) in the cord
- ◯ Yes, there was a combination of two or more of the above issues

If yes please describe

80. What were you told caused your baby's death?

81. If you had an ultrasound during this pregnancy, how many weeks pregnant were you when the last routine ultrasound was done before this baby died? (Do not answer for an ultrasound that was done to confirm your baby's death but rather the one most previous to your baby's death.) Leave blank if you do not remember.

82. What do you believe caused your baby's death?

83. When do you believe this baby died? (Leave blank if you do not know)

Date MM / DD / YYYY

84. When did you first know that your baby was in trouble or had died?

- ☐ I felt a reduction of kicks/movements.
- ☐ I felt kicks/movements stop.
- ☐ I had abdominal pain.
- ☐ I had vaginal bleeding.
- ☐ I had discharge of amniotic fluid/membranes ruptured/water broke.
- ☐ I had a "feeling that something was wrong" but couldn't specify.
- ☐ I had other symptoms - please specify below.
- ☐ I had physical trauma.
- ☐ I was told at an appointment for prenatal care.
- ☐ I was told when admitted for labor.
- ☐ I was told during labor.
- ☐ It was not discovered before my baby was born.
- ☐ I do not remember.

Other - please specify other symptoms

85. What time do you believe your baby died?

- ○ In the morning (6am - 12 noon)
- ○ In the afternoon (12 noon - 6pm)
- ○ In the evening (6pm - 10 pm)
- ○ During the night (10pm - 6am)
- ○ During a daytime nap
- ○ I am not sure.

86. Is there anything else you think is important for us to know about this pregnancy?

87. Please click submit to record your answers.

◯ SUBMIT

New Survey

Thank you for your participation in the STARS Study. Your answers will help us to learn more about stillbirth. If you would like to receive a copy of the summary of the study results when completed and published (this may take 12-18 months) and/or if you would be willing to let us contact you for future stillbirth research projects please click here. Otherwise click 'DONE' exit the study.
